# Improving the normalization of complex interventions: part 2 - validation of the NoMAD instrument for assessing implementation work based on normalization process theory (NPT)

**DOI:** 10.1186/s12874-018-0591-x

**Published:** 2018-11-15

**Authors:** Tracy L. Finch, Melissa Girling, Carl R. May, Frances S. Mair, Elizabeth Murray, Shaun Treweek, Elaine McColl, Ian Nicholas Steen, Clare Cook, Christopher R. Vernazza, Nicola Mackintosh, Samridh Sharma, Gaery Barbery, Jimmy Steele, Tim Rapley

**Affiliations:** 1 Department of Nursing, Midwifery and Health, Northumbria University, Coach Lane, Newcastle-upon-Tyne, NE7 7XA UK; 2 Institute of Health & Society, Newcastle University, Baddiley-Clark Building, Richardson Road, Newcastle-upon-Tyne, NE2 4AX UK; 3 Faculty of Public Health and Policy, London School of Hygiene and Tropical Medicine, 15-17 Tavistock Place, London, WC1H 9SH UK; 4 General Practice and Primary Care, Institute of Health and Wellbeing, University of Glasgow, 1 Horselethill Road, Glasgow, G12 9LX UK; 5 Research Department of Primary Care and Population Health, University College London, Upper Floor 3, Royal Free Hospital, Rowland Hill Street, London, NW3 2PF UK; 6 Health Services Research Unit, University of Aberdeen, 3rd Floor, Health Sciences Building, Foresterhill, Aberdeen, AB25 2ZD UK; 7 School of Law and Business, University of Northumbria, City Campus East 1, Newcastle upon Tyne, NE1 8ST UK; 8 Centre for Oral Health Research, Newcastle University, Framlington Place, Newcastle upon Tyne, NE2 4BW UK; 9 Department of Health Sciences, College of Medicine, Biological Sciences and Psychology, University of Leicester, Centre for Medicine, University Road, Leicester, LE1 7RH UK; 10 International Business and Asian Studies, Griffith University, QLD, Gold Coast, 4222 Australia; 11 Department of Social Work, Education and Community Wellbeing, Northumbria University, Coach Lane, Newcastle-upon-Tyne, NE7 7XA UK

**Keywords:** Normalization process theory, NPT, NoMAD, Implementation process, Questionnaire, Instrument development, Complex interventions

## Abstract

**Introduction:**

Successful implementation and embedding of new health care practices relies on co-ordinated, collective behaviour of individuals working within the constraints of health care settings. Normalization Process Theory (NPT) provides a theory of implementation that emphasises collective action in explaining, and shaping, the embedding of new practices. To extend the practical utility of NPT for improving implementation success, an instrument (NoMAD) was developed and validated.

**Methods:**

Descriptive analysis and psychometric testing of an instrument developed by the authors, through an iterative process that included item generation, consensus methods, item appraisal, and cognitive testing. A 46 item questionnaire was tested in 6 sites implementing health related interventions, using paper and online completion. Participants were staff directly involved in working with the interventions. Descriptive analysis and consensus methods were used to remove redundancy, reducing the final tool to 23 items. Data were subject to confirmatory factor analysis which sought to confirm the theoretical structure within the sample.

**Results:**

We obtained 831 completed questionnaires, an average response rate of 39% (range: 22–77%). Full completion of items was 50% (*n* = 413). The confirmatory factor analysis showed the model achieved acceptable fit (CFI = 0.95, TLI = 0.93, RMSEA = 0.08, SRMR = 0.03). Construct validity of the four theoretical constructs of NPT was supported, and internal consistency (Cronbach’s alpha) were as follows: Coherence (4 items, α = 0.71); Collective Action (7 items, α = 0.78); Cognitive Participation (4 items, α = 0.81); Reflexive Monitoring (5 items, α = 0.65). The normalisation scale overall, was highly reliable (20 items, α = 0.89).

**Conclusions:**

The NoMAD instrument has good face validity, construct validity and internal consistency, for assessing staff perceptions of factors relevant to embedding interventions that change their work practices. Uses in evaluating and guiding implementation are proposed.

**Electronic supplementary material:**

The online version of this article (10.1186/s12874-018-0591-x) contains supplementary material, which is available to authorized users.

## Introduction

Understanding implementation processes is key to ensuring that complex interventions in healthcare are taken up in practice and thus maximize intended benefits for service provision and (ultimately) care to patients [1]. Normalization Process Theory (NPT) [2] provides a framework for understanding how a new intervention becomes part of normal practice. This study aimed to develop and validate an adaptable survey instrument derived from NPT, to be used to improve the implementation of complex healthcare interventions within organisational settings.

We know already that innovations in healthcare are themselves complex [3], and changing practice is difficult. There is a vast literature on implementation in this context and of the need for improvement in how change is implemented [4, 5], however the gap between research evidence and practice remains wide [6, 7]. It is now recognised that improved clinical and health outcomes for healthcare interventions are associated with successful implementation outcomes [1, 8].

Advancements in the measurement of implementation activity have been made recently [9], particularly in relation to the concept of organisational readiness [10–14]. Cook and colleagues [15, 16] have developed a set of measures based on Greenhalgh et al’s model of determinants of diffusion of service innovations [4]. A measurement model based on the Consolidated Framework for Implementation Research (CFIR, [17]) has also been offered, by Liang and colleagues [18]. Hodge and colleagues [19] have recently developed and tested an implementation sustainability measure, and work is also progressing to test a measure of implementation climate [13, 20]. Although these measures show some commonality in the representation of a range of factors affecting implementation success, they differ in their methods of development, and in the extent to which they are based on theoretical underpinnings [21]. Although these advances are promising, improvements in the development and testing of valid and reliable measures for assessing implementation process and outcomes [22] are called for and offer the potential to make important advances in implementation science [21].

This study extends Normalization Process Theory (NPT) [2, 23] towards improving implementation outcomes in the healthcare setting through the use of practical tools to aid implementation ‘work’. NPT provides a framework for understanding how a new intervention becomes part of normal practice, by emphasising the ways in which work must be reconfigured both individually and collectively by multiple stakeholders involved in the work of implementation. The potential for NPT to have far-reaching impact on academic and applied activity to improve the development of complex interventions that are well-placed to become effectively normalized in practice is evident, as demonstrated by the increasing volume of published research that has utilized NPT as a framework for evaluation studies. However, achieving this impact also requires more sophisticated (but simply administered) assessment measures to be developed, tested, and made available to user groups.

To date, few studies have developed quantitative approaches to using NPT (May et al., Review of NPT studies, in preparation). The 16-statement interactive ‘toolkit’ developed in our previous work [24] (available at www.normalizationprocess.org) is presented as a tool to guide service planners, implementers and evaluators in thinking through their implementation problems. However, it is not developed as a research instrument, and nor was it validated for purposes of measuring aspects of implementation activity over time and across settings, as is often the objective of structured assessment in implementation research.

The objectives of NoMAD were thus to: (1) develop a structured instrument for assessing activity related to the normalisation of complex interventions based on NPT, and (2) undertake initial psychometric testing of the instrument in terms of reliability and validity, across a sample of staff involved in the implementation of a range of interventions in different settings. We have reported the development methods and results in detail previously [25]. In this paper, we present the methods and results of the validation phase of the study, and the final NoMAD survey instrument. Guidance on application and adaptation of the instrument for different research and practice activities in provided.

## Methods

A mixed methods approach was undertaken to develop, test and refine the NoMAD instrument using an iterative process. As the development methods are described in detail elsewhere [25], these will be briefly summarised here before detailing the methods used in the main survey validation phase of the project. First, the theoretical foundations for this work are described.

### Theoretical underpinning

NPT [2] is concerned with the generative processes that underpin three core problems: implementation (bringing a practice or practices into action); embedding (when a practice or practices may be routinely incorporated in everyday work of individuals and groups); and integration (when a practice or practices are reproduced and sustained in the social matrices of an organization or institution). There are four generative processes and associated investments (see Table 1).

**Table 1:** NPT Construct definitions

Construct	Definition
Coherence	Sense-making that promotes or inhibits the coherence of a practice to its users. These processes are energized by investments of meaning made by participants
Cognitive participation	Participation that promotes or inhibits users’ enrolment and legitimisation of a practice. These processes are energized by investments of commitment made by participants.
Collective Action	Activity that promotes or inhibits the enacting of a practice by its users. These processes are energized by investments of effort made by participants.
Reflexive monitoring	Practices that promote or inhibit users’ comprehension of the effects of a practice. These processes are energized by investments in appraisal made my participants.

### Item generation and instrument development

Instrument development work focused primarily on generating and testing potential items to reflect each of the four constructs of NPT (coherence, cognitive participation, collective action and reflexive monitoring). An iterative process of instrument development was undertaken using the following methods: theoretical elaboration, item generation and item reduction (team workshops); item appraisal (QAS-99); cognitive testing with complex intervention teams; theory re-validation with NPT experts; and pilot testing of instrument [25]. An overview of the process is presented in Additional file 1.

The NPT construct items utilised a 5 point scale of agreement for response (strongly agree; agree; neither agree nor disagree; disagree; strongly disagree) (Option A response). A set of ‘not relevant’ response options (not relevant to my role; not relevant at this stage; not relevant to [name of intervention]), termed ‘Option B responses’, were included following analysis of cognitive interview data to reflect reasons why participants may be unable to provide a genuine response on the Option A response scale on some items. This was important for validation of the NoMAD tool, in understanding how participants respond to the items.

Three general ‘normalisation’ assessment items were also developed, through a combination of review of existing instruments, workshops, and consensus methods within the research team:

- When you use [intervention], *how familiar* does it feel?
- Do you feel that [intervention] is *currently* a normal part of your work?
- Do you feel that [intervention] *will become* a normal part of your work?

These normalisation assessment items were rated on an 11 point scale (0–10, with appropriate descriptive anchors at 0, 5 and 10). The items were added to the NPT construct items to comprise 46 items for a version of NoMAD that was tested in the main validation study.

### NoMAD validation study

#### Study participants

We sought to recruit at least 300 participants for reliability analysis [26]. A maximum variation approach to sampling of implementation projects was undertaken, seeking diversity in terms of the kinds of interventions being implemented, implementation timelines, and the professionals involved in implementation activity. Inclusion of a site in the project was conditional on sufficient numbers of staff participants to merit a survey approach (minimum of *n* = 20), and access to participants through an appropriate key contact within the site, who could support instrument administration. We sought a minimum of six implementation projects for inclusion.

#### Data collection

Participants in individual sites were identified and accessed through key contacts employed in the sites and invited to participate via email. At each site, instruments were administered either electronically (via SurveyMonkey Inc) or on paper, as advised by site contacts to allow the best chance of maximizing response rates [27]. As most data collection was conducted anonymously and using online methods, written consent was not deemed necessary for this study. As such, consent to participate was provided by individuals on choosing to complete the survey, as explained in the ethical committee approved participant information sheet explaining this procedure for consent, which accompanied all participation invitations. These procedures were approved by the Newcastle University Ethics Committee (Reference number 00555/2012; approval granted 1/09/2012). Site contacts worked with us to adapt the instrument appropriately to their intervention/setting, and issued invitations and reminders of behalf of the NoMAD team. At each site, at least one reminder was issued to all invited participants, within 2 weeks of the initial invitation.

#### Instrument refinement

Exploratory analysis was conducted on the full dataset to inform the retention of items for psychometric testing. Firstly, patterns of item responding were explored through descriptive statistics and frequencies, including checks for floor/ceiling effects, and through correlations amongst the full set of items. These descriptive analyses were combined with consensus methods within the research team, to agree the items retained for the final NoMAD instrument. Item retention was approached by considering each of the 16 sub-constructs as a set, aiming to retain at least one item per sub-construct. A summary table was produced to include descriptive data for each item in terms of:

1. Relative strength of correlations with each other item within the sub-construct
2. Whether the item correlated more highly with *other* sub-constructs within the main construct, than with items within its own sub-construct
3. Whether there were notable correlations between the item and items outside the main construct
4. The strength of correlation with the 3 general normalisation assessment items, and
5. The level of ‘not relevant’ (option B) responding for the item

Table 2 provides an example for the construct of ‘coherence’. Two steps were undertaken towards team consensus regarding retention of items for the final NoMAD survey. Firstly, four team members (TF, TR, MG & CM) independently made item retention selections on the basis of the summary data, with brief justifications for selection and a rating of the difficulty of the decision (0–5, from ‘easy’ to ‘difficult’). Secondly, these judgements were collated and distributed to wider team members for discussion in a full team consensus meeting (to include also EM, FM, ST), where consensus was achieved on the final set of items to be retained. The decision difficulty ratings were used to prioritise the consensus discussions, maximising the input from the wider team. The full set of retained items categorised by the 16 NPT sub-construct domains [2] is presented in Table 3.

**Table 2:** Item retention decision-making process - Example for Coherence

Sub-construct	Item	Strength of correlation with global items (high/mod/low)	Correlation with other s-c items?	Higher correlation with items in *other* s-c?	Noteworthy correlation with other construct items?	Level of option B responding? %
Differentiation1	I can distinguish the [intervention] from usual ways of working	Best for ‘will become’ (0.35), v low for other items.	Correlation of .70 with each other.	No	No	8.9
Differentiation2	I can see how the [intervention] differs from usual ways of working	Similar to diff1 (0.29 highest).		No	No	8.1
Communal spec1	Staff in this organisation have a shared understanding of the purpose of the [intervention]	Approx 0.30 across all 3.	Moderate (0.59)	No	IndivSpec1 (0.50)	4.2
Communal spec2	Staff in this organisation have shared expectations about the likelihood of the success of the [intervention]	Low. Highest is 0.26 for ‘will become’.		No	Activation2 (0.51). Correlations with appraisal items in RefMon (0.46–.50).	5.0
Individual spec1	I understand what tasks the [intervention] requires of me	Mod (0.40–0.47)	Moderate (0.67)	No	Internalization items (0.60 approx). CommSpec1 (0.50). Workability items (0.48&0.50). Legitimation items in CP (0.45 & 0.46)	3.3
Individual spec2	I understand how the [intervention] affects the nature of my own work	Mod (0.38–0.41)		No	Similar as for IndSpec1.	3.1
Internalization1	I can see the potential value of the [intervention] for my work	Varies across 3 items. Mod (0.48) for ‘will become’, 0.34 for ‘is normal’, 0.29 for familiarity.	High (0.83)	No	IndSpec2, 0.61. Legit1, 0.61. Activ1, 0.65. Mod with appraisal items (RefMon), approx. 0.50–0.58 (IndApprais3).	2.3
Internalization2	I can see the worth of the [intervention] for me	Similar for items 1&2 (0.31 & 0.35). Mod (0.49) for ‘will become’.			As for internalisation1	2.5

**Table 3:** Retained items by NPT sub-construct domains

Construct	Sub-Construct	Items
*Coherence*	*Differentiation*	I can see how the [intervention] differs from usual ways of working
	*Communal specification*	Staff in this organisation have a shared understanding of the purpose of this [intervention]
	*Individual specification*	I understand how the [intervention] affects the nature of my own work
	*Internalization*	I can see the potential value of the [intervention] for my work
*Cognitive Participation*	*Initiation*	There are key people who drive the [intervention] forward and get others involved
	*Legitimation*	I believe that participating in the [intervention] is a legitimate part of my role
	*Enrolment*	I’m open to working with colleagues in new ways to use the [intervention]
	*Activation*	I will continue to support the [intervention]
*Collective Action*	*Interactional workability*	I can easily integrate the [intervention] into my existing work
	*Relational integration*	The [intervention] disrupts working relationships
	*Relational integration*	I have confidence in other people’s ability to use the [intervention]
	*Skill set workability*	Work is assigned to those with skills appropriate to the [intervention]
	*Skill set workability*	Sufficient training is provided to enable staff to use the [intervention]
	*Contextual Integration*	Sufficient resources are available to support the [intervention]
	*Contextual integration*	Management adequately support the [intervention]
*Reflexive Monitoring*	*Systemisation*	I am aware of reports about the effects of the [intervention]
	*Communal appraisal*	The staff agree that the [intervention] is worthwhile
	*Individual appraisal*	I value the effects the [intervention] has had on my work
	*Reconfiguration*	Feedback about the [intervention] can be used to improve it in the future
	*Reconfiguration*	I can modify how I work with the [intervention]

#### Psychometric analysis

Psychometric analysis was conducted on the pooled dataset. Construct validity was explored through examination of the bivariate correlations between all possible pairs of construct items. We would expect items from the same construct to be more highly correlated with each other than with items measuring different constructs. Internal consistency was assessed using Cronbach’s alpha. A value of > = 0.7 is usually taken as indicative of adequate internal consistency.

Data were subject to confirmatory factor analysis (CFA) which sought to investigate if the theoretically derived model approximated to the data. Since variables were judged to be unidimensional, parcelling was undertaken in order to maximise reliability [28] communality [29] and the value of the fit statistics [30] and to transform the ordinal data into a closer approximation to continuous data [31]. To create the parcels, the item-to-construct balance parcelling methodology was used [32].

Model fit was assessed by consulting the Tucker Lewis Index (TLI), Comparative Fit Index (CFI), Root Mean Square Error of Approximation (RMSEA) and the Standardised Root Mean Square Residual (SRMR). The primary aim was to achieve fit across all relevant indices. Specifically, we ideally sought a TLI and CFI of at least 0.90 and ideally 0.95 [33], an RMSEA below 0.07 [34] and an SRMR below 0.08. However in line with Hu and Bentler’s [35] guidelines a model acceptably approximates the data if a TLI or CFI of around 0.95 or an RMSEA up to around to 0.06 is observed alongside an SRMR up to around 0.08 [35]. The confirmatory factor analysis was undertaken within *M*plus.

#### Ethics

Ethical approval for this study was granted by the University of Newcastle Ethics Committee (Reference number 00555/2012; approval granted 1/09/2012).

## Results

### Response

Six implementation projects contributed data for instrument validation, representing a variety of professional roles in relation to the interventions that were being implemented (for example, clinical, administrative, managerial, and other professionals in non-health contexts).

Across these interventions, a total of 831 surveys were submitted. An overall response rate cannot be calculated as the denominator cannot be determined for one site (S6). Excluding S6, the response rate is 35% (495/1423). A breakdown of response data by site is provided in Table 4. Out of 831, 522 participants (63%) responded to one or more of the 43 NoMAD construct items, with 413 participants (50% of total sample) responding to all items. Excluding ‘Option B’ (‘not relevant’) responses, a total of 248 participants provided a likert scale (5 pt) response for all 43 construct items. Response rates were variable across items. Non-response at the individual item level ranged from 0.6% (*n* = 3) to 12% (*n* = 61). Response rates to option A (item deemed relevant for a likert rating) ranged from 75% (*n* = 389) to 97% (*n* = 508) across items.

**Table 4:** Response rates and item completion

Dataset	Mode of administration	Invited	Responded	RR Total N/ Invited	Number of items completed		Total completing 1–43 items
					1–42 items	All 43 items	
S1: Digital Health	Electronic + paper	231	67	29%	16	37	53
S2: Smoking cessation	Paper	100	21	21%	3	18	21
S3: Patient self-management	Electronic	400	91	23%	26	51	77
S4: Oral Health Risk Assessement	Paper with personal approach Electronic (5th years)	297	229	77%	26	194	220
S5: System level IT	Electronic	395	87	22%	14	53	67
S6: Sports programme	Electronic – not targeted	Unknown	336	Unknown	24	60	84
Total/ Overall		>1423^a^	831	35%^a^	109	413	522

^a^Excluding S6

### Participant characteristics

Information about participants’ professional roles is provided in Tables 5 and 6.

**Table 5:** Description of study participants’ roles (*N* = 522) (% (n)

Site 1: Digital health record (*N* = 53)		Site 2: Smoking cessation (*N* = 21)		Site 3: Patient self-management tool (*N* = 77)		Site 5: Technology implementation (*N* = 67)		Site 6 (*N* = 84)	
Professional Role		Professional Role		Professional Role		Professional Role		Organisation level	
Health visitor	76 (40)	Community Midwife	67 (14)	GP	35 (27)	Consultant	24 (16)	National Sporting organisation	6 (5)
Nursery nurse	6 (3)	Hospital midwife	24 (5)	Hospital Doctor	5 (4)	Trainee Doctor	5 (3)	State Sports Organisation (SSO)	14 (12)
Team leader	2 (1)	Stop smoking advisor	5 (1)	Consultant	4 (3)	Nurse Band 5	19 (13)	League	11 (9)
Student health visitor	9 (5)	Stop smoking clerical/admin	0 (0)	Occupational Therapist	1 (1)	Nurse Band 6	22 (15)	Club	61 (51)
Family nurse partnership	8 (4)	(missing)	5 (1)	Physiotherapist	21 (16)	Nurse Band 7	15 (10)	Other	8 (7)
(missing)	0 (0)			Practice Nurse	17 (13)	Nurse Band 8	2 (1)		
				Hospital Nurse	9 (7)	Admin clerical - secretary	2 (1)	Role in Organisation	
				School Nurse	1 (1)	Manager	5 (3)	Administrator (Paid)	20 (17)
				Dietician	4 (3)	Physiotherapist	2 (1)	Administrator (Volunteer)	39 (33)
				Pharmacist	3 (2)	Dietician	3 (2)	Coach	31 (26)
				missing	0 (0)	Pharmacist	2 (1)	Other	10 (8)
						Other	2 (1)		
Main role in relation to the intervention		Main role in relation to the intervention		Geographic region (role not elicited)		Main role in relation to the intervention		Main role in relation to the intervention	
Champion	13 (7)	I am involved in managing or overseeing [name] intervention	19 (4)	Region 1	55 (42)	I am involved in managing, overseeing or being a clinician	28 (19)	I’m involved in implementing [sports programme]	57 (48)
Promoter	87 (46)	I am involved in delivering the [name] intervention	62 (13)	Region 2	34 (26)	I will be involved in working with the system of [name]	69 (46)	I’m involved in managing/overseeing [programme]	43 (36)
		missing	19 (4)	Region 3	12 (9)	Missing	3 (2)		
Years worked in the Trust		Years worked in the Trust		Years worked in the Trust		Years worked in the Trust			
< 1 year	15 (8)	< 1 year	10 (2)	< 1 year	0 (0)	< 1 year	10 (7)		
1–2 years	15 (8)	1–2 years	0	1–2 years	1 (1)	1–2 years	15 (10)		
3–5 years	9 (5)	3–5 years	5 (1)	3–5 years	7 (5)	3–5 years	19 (13)		
6–10 years	21 (11)	6–10 years	5 (1)	6–10 years	10 (8)	6–10 years	30 (20)		
11–15 years	21 (11)	11–15 years	24 (5)	11–15 years	14 (11)	11–15 years	15 (10)		
> 15 years	19 (10)	> 15 years	52 (11)	> 15 years	68 (52)	> 15 years	10 (7)		
missing	0	missing	5 (1)	missing	0

**Table 6:** Site 4 Oral health risk assessment (Students and clinicians) descriptives

Site 4: Oral health risk assessment Student dentists (*N* = 189)		Site 4: Oral health risk assessment Clinicians (*N* = 31)	
Year of study		Professional Role	
3rd Year	40 (77)	Clinical Fellow	13 (4)
4th Year	41 (77)	Senior lecturer	7 (2)
5th Year	20 (37)	Professor	10 (3)
		Associate Clinical Lecturer	55 (17)
		Clinical Trainer	7 (2)
		StR/SpR	7 (2)
		Missing	3 (1)
Main role in relation to the intervention		Main role in relation to the intervention	
		I oversee others delivering [risk assessment] scores	Yes: 84 (26) No: 3 (1) DA: 13 (4)
I directly deliver the [risk assessment] scores to patients	Yes: 94 (177) No: 6 (12)	I directly deliver [risk assessment] scores to patients	Yes: 42 (13) No: 42 (13) DA: 16 (5)
I use and deliver [risk assessment] scores in another setting	Yes: 6 (12 No: 74 (139) DA: 20 (38)	I use and deliver [risk assessment] scores in another setting	Yes: 23 (7) No: 58 (18) DA: 19 (6)
		Years worked in the [Dental Hospital]	
		< 1 year	10 (3)
		1–2 years	10 (3)
		3–5 years	19 (6)
		6–10 years	16 (5)
		11–15 years	13 (4)
		> 15 years	32 (10)

### Relationships amongst NPT constructs

The NoMAD items were developed to represent pre-defined theoretical constructs. On this basis, we expected items within the four theoretical constructs (coherence, cognitive participation, collective action, and reflexive monitoring) to be more strongly related to each other, than to items in other constructs. Inspection of bivariate correlation matrices generally confirmed the expected pattern of relationships [available from authors on request].

Bivariate correlations between the NPT construct measures are shown in Table 7 revealing a moderate level of correlation for summated scores within the construct domains. This suggests that Coherence and Cognitive Participation are the most highly correlated (*r* = .68), and Reflexive Monitoring and Collective Action (*r* = .49) are the two constructs that are least correlated.

**Table 7:** Correlations between construct measures

	Coherence	Cognitive Participation	Collective Action
Coherence	1		
Cognitive Participation	**.68** (*n* = 512)	1	
Collective Action	**.55** (*n* = 454)	**.54** (*n* = 456)	1
Reflexive Monitoring	**.60** (*n* = 427)	**.59** (*n* = 428)	**.49** (*n* = 423)

Pearson Correlation, all sig. (2 tailed) < .000

Correlations between the construct measures scores and the overall normalisation score, with the general assessment items are shown in Table 8. Correlations were low to moderate. Of the three general assessment items, the construct measures appear to relate most strongly to perceptions that a new intervention will become a normal part of work.

**Table 8:** Correlations between construct measures and general assessment items

	How familiar?	Normal part of your work?	Will it become normal?
Coherence	**.35** (*n* = 448)	**.43** (*n* = 447)	**.54** (*n* = 443)
Cognitive Participation	**.25** (*n* = 431)	**.30** (*n* = 430)	**.47** (*n* = 426)
Collective Action	**.41** (*n* = 409)	**.48** (*n* = 408)	**.45** (*n* = 404)
Reflexive Monitoring	**.26** (*n* = 388)	**.28** (*n* = 387)	**.40** (*n* = 383)
Normalisation score	**.41** (*n* = 417)	**.48** (*n* = 416)	**.58** (*n* = 412)

Pearson Correlation, all sig. (2 tailed) < .0

### Factor structure

The NoMad items were entered into a CFA to replicate the theoretical model. This item level CFA was run using the Weighted Least Squares Means and Variances (WLSMV) estimator which was considered appropriate given that the data were technically ordinal [36, 37]. The model showed reasonable fit (CFI = 0.91, TLI = 0.90, RMSEA = 0.09). Based on the factor loadings, the items were parcelled using the item-to-construct balance technique (see Table 9 for parcel composition). The CFA was then rerun using the ML estimator since the parcelled data more closely approximated continuous data. The resultant model showed a level of fit (CFI = 0.93, TLI = 0.89, RMSEA = 0.11, SRMR = 0.05) just short of acceptable [35]. The modification indices were therefore consulted and on this basis two correlated errors were modelled. The resultant model achieved acceptable fit (CFI = 0.95, TLI = 0.93, RMSEA = 0.08, SRMR = 0.03). Table 9 presents the standardized factor loadings.

**Table 9:** Confirmatory Factor Analysis (CFA) Parcel composition & Standardised indicator loadings

Parcel	Item	F1	F2	F3	F4
Coherence parcel 1	I can see the potential value of the [intervention] for my work I can see how the [intervention] differs from usual ways of working	.74			
Coherence parcel 2	Staff in this organisation have a shared understanding of the purpose of this [intervention] I understand how the [intervention] affects the nature of my own work	.67			
Cognitive participation parcel 1	There are key people who drive the [intervention] forward and get others involved I will continue to support the [intervention]		.84		
Cognitive participation parcel 2	I believe that participating in the [intervention] is a legitimate part of my role I’m open to working with colleagues in new ways to use the [intervention]		.86		
Collective action parcel 1	The [intervention] disrupts working relationships I can easily integrate the [intervention] into my existing work			.67	
Collective action parcel 2	Work is assigned to those with skills appropriate to the [intervention] Sufficient resources are available to support the [intervention]			.57	
Collective action parcel 3	I have confidence in other people’s ability to use the [intervention] Sufficient training is provided to enable staff to use the [intervention] Management adequately support the [intervention]			.66	
Reflexive monitoring parcel 1	I am aware of reports about the effects of the [intervention] I value the effects the [intervention] has had on my work				.55
Reflexive monitoring parcel 2	The staff agree that the [intervention] is worthwhile Feedback about the [intervention] can be used to improve it in the future I can modify how I work with the [intervention]				.70

### Internal consistency of NPT construct subscales

Cronbach’s alpha was calculated for each of the four NPT construct groupings. Coherence consists of four items (α = 0.71); cognitive participation includes four items (α = 0.81); collective action comprises seven items (α = 0.78); and reflexive monitoring contains five items (α = 0.65). The normalisation scale overall (comprising items across all four constructs), was highly reliable (20 items, α = 0.89). Further information about item-total statistics is available from the authors.

## Discussion

This paper presents NoMAD as a theoretically derived instrument for assessing implementation processes from the perspective of individuals involved in implementation activity. This further advances emerging work on the measurement of implementation processes [20–22, 36–40], by offering a theory-based measurement tool underpinned by Normalization Process Theory (NPT).

To our knowledge, NoMAD represents the first systematic development and validation of a structured assessment tool based on the theoretical constructs of NPT [41]. NPT proposes that the embedding of new practices requires participants involved in the process to engage in work across four construct domains of coherence, cognitive participation, collective action and reflexive monitoring. The Confirmatory Factor Analysis (CFA) of the items we retained using descriptive data and consensus methods, supported this proposed theoretical structure. Tests of internal consistency supported the use of these items either as an overall measure of ‘normalisation’ (20 items, α = 0.89), or as four construct measures (ranging from α = 0.65–0.81). Except for reflexive monitoring, all constructs achieved the desired threshold of ≥0.07. Together with the moderate correlations between the four construct measures, the data supports the proposition that these are related but conceptually distinct domains within the theory. NoMAD has good face validity, and construct validity. NoMAD compares favourably against reviews of other implementation and research utilization measures, which generally rate the psychometric properties of instruments as poor to modest [22, 40], and show few (if any) measures to meet the full range of psychometric criteria being assessed.

As a structured instrument, NoMAD offers scope to better understand the theoretical mechanisms of NPT by exploring, statistically, the relative importance of the NPT constructs in achieving sustained practice changes. NPT does not currently ascribe relative weightings to the importance of different construct domains for achieving the normalisation of a new practice, and the growing body of qualitative research framed by NPT [42] would suggest that the importance of the construct domains will vary according to the unique combination of intervention, context and human factors involved in the target practice change. A study by Jacobs and colleagues [20] to test a measure of Implementation Climate [13] found that although a common factor structure was supported in different organisational settings that was consistent with the theoretical specification (subscales on whether use of an innovation is *rewarded*, *supported* or *expected* within their organisation), the relevant strength of factor loadings of individual items varied across the settings. In their study, the settings differed in the extent to which participants worked primarily independently versus collaboratively, in ways that made sense in relation to the relative contexts. They concluded that indeed ‘context matters’ in measuring implementation climate, and argued that CFA models can still advance theory and knowledge of implementation even if they cannot be reasonably expected to fit other studies or contexts [20]. As authors of NoMAD, we wish to see further validation of the instrument in different settings, but caution that interpretation of results of different applications of NoMAD will need to be informed by understanding of important contextual features of the study context, often derived from qualitative work.

We suggest therefore that NoMAD should be viewed as a ‘pragmatic measure’ of implementation [43] and encourage users to apply it flexibly to their implementation research and practice needs. Consistent with Glasgow and Riley’s [43] call for ‘pragmatic measures’ that balance psychometric considerations against the requirements of implementation in real life contexts, we believe that developing NoMAD with equal emphasis on (theoretical) content and face validity, and respondent usability (through cognitive interviews) has resulted in a measure that meets their required criteria of being important to stakeholders, low burden to respondents, and actionable (in that it can point to problems that can be further investigated or addressed in practice). Further, NoMAD also meets the recommended criteria of being broadly applicable (to a range of settings), unlikely to cause harm, related to theory, and psychometrically strong. A further required criterion of ‘sensitivity to change’ (often termed ‘responsiveness’ [39]) will be assessed in further validation work where multiple time point measures can be taken and compared. A review of implementation measures for community and public health settings found that all measures tested for responsiveness (7/51 measures reviewed), showed a minimum of moderate effect size (criterion of 0.5), suggesting the capacity of such measures to be responsive to change.

In practical terms then, we offer NoMAD as an adaptable ‘bank of items’ that may be used flexibly by researchers or implementers [Additional file 2]. We anticipate that some will wish to use NoMAD as a complete instrument as presented here, with minimal adaptation besides adjustments for appropriate contextualisation of the survey to the target practice change. For these purposes, the validation data provided in this paper is important for the scientific endeavour of their work, as will other authors’ validation studies be important for further development of our work with NoMAD and with NPT. However in other applications of NoMAD, it may be that only certain items (or sets of items) may be relevant for use, and/or at particular stages of an implementation process. For example some items may be less useful in the very early stages of implementation, when no one has actually worked with the intervention or the intervention is not fully developed. The wording of the items may need to be altered, for example, for more anticipatory assessments. The extent to which the validation data reported in this paper can be expected to apply to future uses of NoMAD will therefore vary with the level of adaption made to the items, and will be a matter of the users’ own judgement. The NPT website (http://www.normalizationprocess.org) provides advice for how NoMAD may be used and adapted, but this is merely guidance.

Consistent with this position, it is not possible to prescribe a formulaic process for scoring or combining items, or for the interpretation of the results that NoMAD generates, for all settings. In its simplest, most descriptive form, the underlying assumption of NoMAD is that more positive ratings by respondents of the implementation processes represented in each of the items are suggestive of higher potential for the practice to normalise. However this remains open to further assessment, and interpretation of results from the items used will always need to be undertaken in context. For example, whether an intervention ‘differs from usual ways of working’ may be of benefit to the implementation process in some contexts, but more problematic in others. For this reason, we present NoMAD as four sets of construct items, with reliability and validity data, and do not offer specific instructions for scoring or creating construct measures. Where assessments at the level of the construct are merited, items within the construct may be averaged to create ‘scores’ that may be compared amongst constructs, or between groups, or sites, if appropriate to the objective of the investigation.

Further validation of NoMAD is required to assess how the NoMAD constructs relate to measures that are both theoretically similar (convergent validity) and conceptually distinct (discriminant validity). Appropriate measures for these purposes were not available at the time NoMAD was being developed, but a diverse range of implementation success indicators are now rapidly emerging and subject to critical review [39]. Existing implementation measures vary greatly but include tools that emphasise behavioural dimensions (eg. Theoretical Domains Framework) [44–46], organisational readiness [14, 47], implementation climate [20], research utilization in practice [39], and more focused constructs such as ‘implementation leadership’ [48]. It is likely that other measures more closely aligned with the focus of NPT will follow. Although defining and measuring concepts relating to implementation outcomes, or ‘success’ still remains complex [22, 49] due to the variety of theories and frameworks, studies that incorporate testing of sets of multiple measures will enhance our understanding of implementation process and outcomes. NoMAD should also be tested for criterion-related validity, to assess whether the construct measures are associated with implementation outcomes as proposed by NPT. These assessments can be undertaken in well designed longitudinal studies, that include robust implementation outcome measures [22]. We are currently undertaking such work in a range of contexts, including the ImpleMentAll study (http://www.implementall.eu/), which will use measures including NoMAD items,^1^ to assess the outcome of tailored implementation interventions in various settings across eight countries.

This study provides support for NoMAD as an adaptable set of construct measures based on NPT. Key strengths include the underlying theoretical basis, and the iterative approach to item development and retention that prioritised construct validity, and usability from the perspective of a range of professionals involved in implementing complex interventions, in healthcare and other settings (education and sport). Current limitations however include validation work that is not yet undertaken, in terms of test-retest reliability, and convergent, discriminant and criterion-related validity. NoMAD is also limited to the frame of reference that NPT itself offers – a focus on the work that people undertake when implementing changes in practice, from the perspective of those involved in this work. Like any theory, or derivative instrument, its application must be appropriate to its frame of focus. NPT asks us to observe and understand social action, at an individual and collective level. As a self-report measure of individual’s perceptions of these processes, a fuller understanding of the embedding of a practice in any given setting is likely to require a combination of approaches that include quantitative and qualitative investigations. However, where larger scale implementation studies aim to compare implementation progress *across multiple sites* involved in an implementation project, and/or activity *over time*, NoMAD offers an appropriate tool to assess this. In some settings, NoMAD may be used as a diagnostic instrument, enabling data collection for local adaptation and improvement.

## Conclusion

NoMAD provides a tool based on Normalization Process Theory (NPT), for the structured assessment of the work of implementation from the perspective of staff involved in implementing changes in practice. The key result of this study is the NoMAD instrument, along with validation data concerning statistical properties and other information that can be used to guide the application of the measures across different settings and for different purposes (designing, monitoring, and for trouble-shooting interventions and their implementation). As such, NoMAD should be viewed as a further tool in the NPT ‘toolkit’ (http://www.normalizationprocess.org), alongside the interactive 16 item tool [24] that was designed specifically for academic and non-academic users of NPT to think through their implementation problems, more as a ‘sensitizing’ device than as a validated tool for measuring implementation process and outcomes. We anticipate that although NoMAD is itself an important product of the study, the generation (and dissemination) of experience and knowledge in the application of these measures to real problems of implementation of complex health interventions in diverse settings will be the key to improving the design and implementation of interventions that are ultimately intended to benefit recipients of health care services.

## Additional files

Additional file 1: Overview of instrument development process. Provides process map of methods and data collected to develop and refine the NoMAD instrument. (DOCX 119 kb)

Additional file 2: Full NoMAD survey with adaptation guidance. Provides a copy of the full NoMAD survey instrument with guidance for adaptation. (DOCX 187 kb)

## Abbreviations

CFA: Confirmatory Factor Analysis
CFI: Comparative Fit Index
CFIR: Consolidated Framework for Implementation Research
NoMAD: Normalisation Measure Development (study name)
NPT: Normalization Process Theory
QAS-99: Question Appraisal System
RMSEA: Root Mean Square Error of Approximation
SRMR: Standardised Root Mean Square Residual
TLI: Tucker Lewis Index

## Footnotes

1 The factor structure of NoMAD items from an existing dataset on e-mental health will be used to develop a version of NoMAD appropriate for the analysis of the ImpleMentAll study dataset.
