# Supplementary figures and images for: Improving the normalization of complex interventions: part 2 - validation of the NoMAD instrument for assessing implementation work based on normalization process theory (NPT)
Source: BMC Med Res Methodol. 2018 Nov 15;18:135. doi: 10.1186/s12874-018-0591-x (PMC6238372; doi:10.1186/s12874-018-0591-x)

Additional file 1

Overview of NoMAD development methods

Source: paper 1


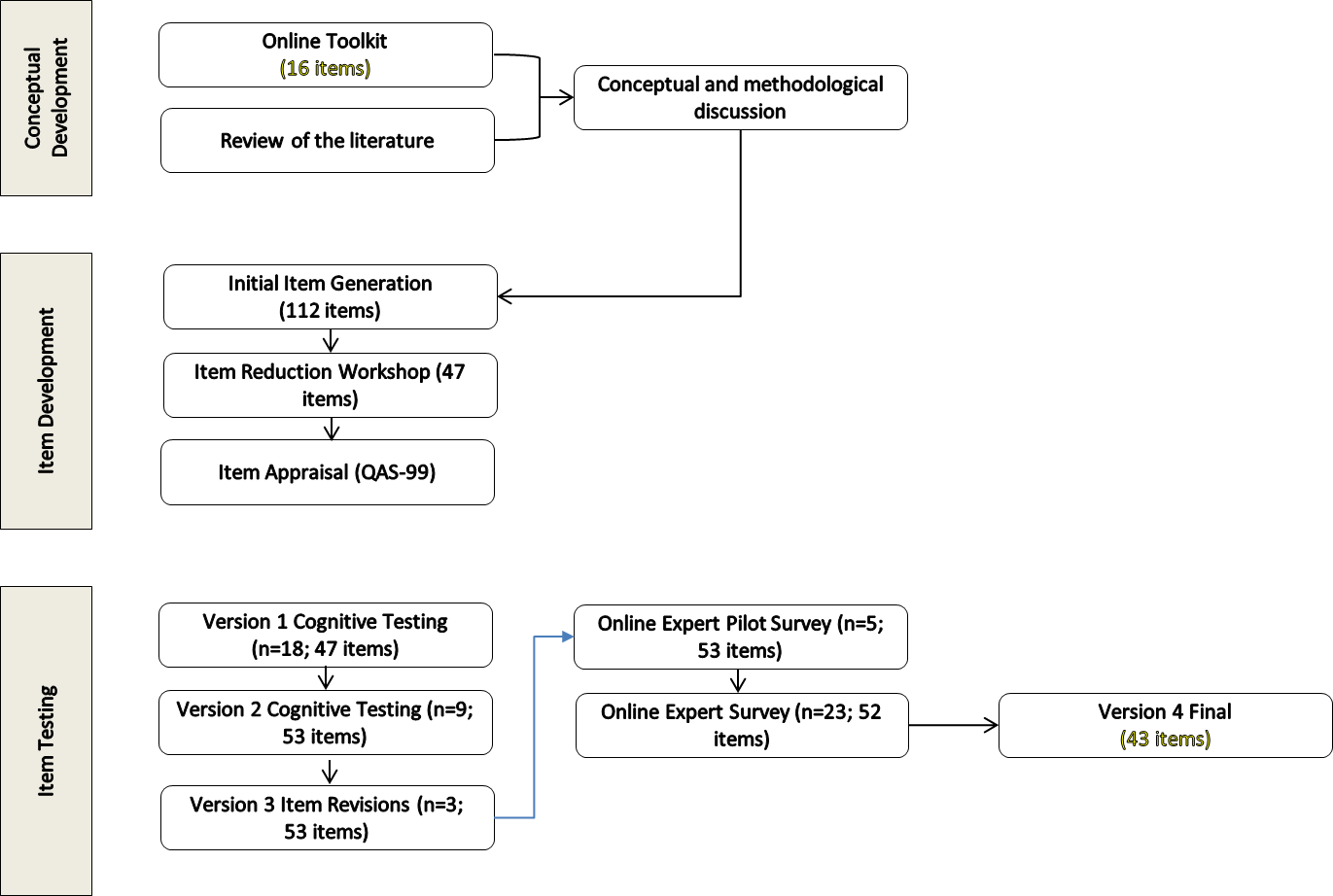

Supplement: Supplementary file 1 — Overview of instrument development process. Provides process map of methods and data collected to develop and refine the NoMAD instrument. (DOCX 119 kb) [file 12874_2018_591_MOESM1_ESM.docx]
