# Supplementary material for: Improving the normalization of complex interventions: part 2 - validation of the NoMAD instrument for assessing implementation work based on normalization process theory (NPT)
Source: BMC Med Res Methodol. 2018 Nov 15;18:135. doi: 10.1186/s12874-018-0591-x (PMC6238372; doi:10.1186/s12874-018-0591-x)
Supplement: Supplementary file 2 — Full NoMAD survey with adaptation guidance. Provides a copy of the full NoMAD survey instrument with guidance for adaptation. (DOCX 187 kb) [file 12874_2018_591_MOESM2_ESM.docx]

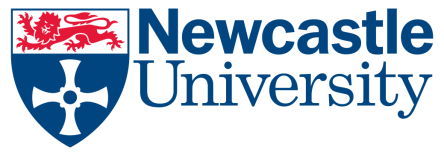

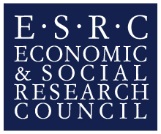


**Please cite as:** Finch, T.L., Girling, M., May, C.R., Mair, F.S., Murray, E., Treweek, S., Steen, I.N., McColl, E.M., Dickinson, C., Rapley, T. (2015). NoMad: Implementation measure based on Normalization Process Theory. [Measurement instrument]. Retrieved from <http://www.normalizationprocess.org>.

**Survey Instructions**

**[YOU MUST ADAPT THE TEXT FOR OWN STUDY – REMEMBER TO REPLACE [the intervention] WITH YOUR OWN TERM]**

**This survey is designed to help get a better understanding of how to apply and integrate new technologies and complex interventions in health care.**

This survey asks questions about the implementation of **[the intervention]**. We understand that people involved with **[the intervention]** have different roles, and that people may have more than one role.

From the statements below please choose an option that best describes ***your main role*** in relation to **[the intervention]*:***

- **I am involved in managing or overseeing [the intervention] 🞏 [THIS LIST MAY NOT BE NEEDED]**
- **I am involved in delivering [the intervention]**  **🞏**

For this survey, please answer all the statements from the perspective of this role. Depending on your role or responsibilities in [the intervention], some statements may be more relevant than others.

The survey is in [X NUMBER] parts. Part A asks some brief questions about yourself and your role. Part B includes three general questions about **[the intervention] [NB: ADDITIONAL QUESTIONS CAN BE ADDED]**. Part C contains a set of more detailed questions about **[the intervention]**. For each statement in Part C, there is the option to agree or disagree with what is being asked **(OPTION A).** However, if you feel that the statement is not relevant to you, there are also options to tell us why **(OPTION B). [NB: ADAPT & ADD TO AS APPROPRIATE]**

Please take the time to decide which answer **best suits your experience for each statement and tick the appropriate circle**

*Development of this survey was funded by the Economic and Social Research Council; Study Grant RES-062-23-3274. The core NPT items (20 construct items & 3 normalisation items) are Copyright © Newcastle University 2015.*

| **Part A: About yourself [ADD APPROPRIATE ROLE OR OTHER QUESTIONS FOR DESCRIBING YOUR PARTICIPANTS]** | | | | | | | | | | | |  |  |
| --- | --- | --- | --- | --- | --- | --- | --- | --- | --- | --- | --- | --- | --- |
| 1. **How many years have you worked for this [name of organisation/department]*? (If your Trust has merged with another or changed its name, please include in your answer all the time you have worked with this Trust and its predecessors)*** | | | | | | | | | | | | | |
|  | | Less than one year |  | 1-2 years |  | 3-5 years |  | 6-10 years |  | 11-15 years |  | More than 15 years |  |
| 1. **How would you describe your professional job category? [WE ADVISE SPECIFYING APPROPRIATE CATEGORIES]** | | | | | | | | | | | | | |

|  |
| --- |

| **Part B: General questions about the intervention [THESE CAN BE ADAPTED, DROPPED AND/OR ADDED TO AS APPROPRIATE]** |
| --- |
|  |

| When you use [the intervention], how familiar does it feel? | | | | | | | | | | | | |
| --- | --- | --- | --- | --- | --- | --- | --- | --- | --- | --- | --- | --- |
| Still feels very new | | | |  | | | | | **Feels completely familiar** | | | |
|  | | | |  | | | | |  | | | |
| 0 | 1 | 2 | 3 | | 4 | 5 | 6 | 7 | | 8 | 9 | 10 |
| Do you feel [the intervention] is currently a normal part of your work? | | | | | | | | | | | | |
| Not at all | | | | **Somewhat** | | | | | **Completely** | | | |
|  | | | |  | | | | |  | | | |
| 0 | 1 | 2 | 3 | | 4 | 5 | 6 | 7 | | 8 | 9 | 10 |
| Do you feel [the intervention] will become a normal part of your work? | | | | | | | | | | | | |
|  | | | | | | | | | | | | |
| Not at all | | | | **Somewhat** | | | | | **Completely** | | | |
|  | | | |  | | | | |  | | | |
| 0 | 1 | 2 | 3 | | 4 | 5 | 6 | 7 | | 8 | 9 | 10 |

| **Part C: Detailed questions about the intervention [WE ADVISE MINOR ADAPTATION ONLY AS REQUIRED TO MAKE SENSE]** |
| --- |

**For each statement please select an answer that best suits your experience using Option A. If the statement is not relevant to you please select an answer from Option B.**

|  | | **Option A** | | | | |  | **Option B** | | |
| --- | --- | --- | --- | --- | --- | --- | --- | --- | --- | --- |
| **Section C1** | | **Strongly Agree** | **Agree** | **Neither agree nor disagree** | **Disagree** | **Strongly disagree** |  | **Not relevant to my role** | **Not relevant at this stage** | **Not relevant to the intervention** |
| **1.** | **I can see how [the intervention] differs from usual ways of working** |  |  |  |  |  |  |  |  |  |
| **2.** | **Staff in this organisation have a shared understanding of the purpose of [the intervention]** |  |  |  |  |  |  |  |  |  |
| **3.** | **I understand how [the intervention] affects the nature of my own work** |  |  |  |  |  |  |  |  |  |
| **4.** | **I can see the potential value of [the intervention] for my work** |  |  |  |  |  |  |  |  |  |

**For each statement please select an answer that best suits your experience using Option A. If the statement is not relevant to you please select an answer from Option B.**

|  | |  |  | **Option A** | |  |  | **Option B** | | |
| --- | --- | --- | --- | --- | --- | --- | --- | --- | --- | --- |
| **Section C2** | | **Strongly Agree** | **Agree** | **Neither agree nor disagree** | **Disagree** | **Strongly disagree** |  | **Not relevant to my role** | **Not relevant at this stage** | **Not relevant to the intervention** |
| **1.** | **There are key people who drive [the intervention] forward and get others involved** |  |  |  |  |  |  |  |  |  |
| **2.** | **I believe that participating in [the intervention] is a legitimate part of my role** |  |  |  |  |  |  |  |  |  |
| **3.** | **I’m open to working with colleagues in new ways to use [the intervention]** |  |  |  |  |  |  |  |  |  |
| **4.** | **I will continue to support [the intervention]** |  |  |  |  |  |  |  |  |  |

**For each statement please select an answer that best suits your experience using Option A. If the statement is not relevant to you please select an answer from Option B.**

|  | | **Option A** | | | | |  | **Option B** | | |
| --- | --- | --- | --- | --- | --- | --- | --- | --- | --- | --- |
| **Section C3** | | **Strongly Agree** | **Agree** | **Neither agree nor disagree** | **Disagree** | **Strongly disagree** |  | **Not relevant to my role** | **Not relevant at this stage** | **Not relevant to the intervention** |
| **1.** | **I can easily integrate [the intervention] into my existing work** |  |  |  |  |  |  |  |  |  |
| **2.** | **[The intervention] disrupts working relationships** |  |  |  |  |  |  |  |  |  |
| **3.** | **I have confidence in other people’s ability to use [the intervention]** |  |  |  |  |  |  |  |  |  |
| **4.** | **Work is assigned to those with skills appropriate to [the intervention]** |  |  |  |  |  |  |  |  |  |
| **5.** | **Sufficient training is provided to enable staff to implement [the intervention]** |  |  |  |  |  |  |  |  |  |
| **6.** | **Sufficient resources are available to support [the intervention]** |  |  |  |  |  |  |  |  |  |
| **7.** | **Management adequately supports [the intervention]** |  |  |  |  |  |  |  |  |  |

**For each statement please select an answer that best suits your experience using Option A. If the statement is not relevant to you please select an answer from Option B.**

|  | | **Option A** | | | | |  | **Option B** | | |
| --- | --- | --- | --- | --- | --- | --- | --- | --- | --- | --- |
| **Section C4** | | **Strongly Agree** | **Agree** | **Neither agree nor disagree** | **Disagree** | **Strongly disagree** |  | **Not relevant to my role** | **Not relevant at this stage** | **Not relevant to the intervention** |
| **1.** | **I am aware of reports about the effects of [the intervention]** |  |  |  |  |  |  |  |  |  |
| **2.** | **The staff agree that [the intervention] is worthwhile** |  |  |  |  |  |  |  |  |  |
| **3.** | **I value the effects that [the intervention] has had on my work** |  |  |  |  |  |  |  |  |  |
| **4.** | **Feedback about [the intervention] can be used to improve it in the future** |  |  |  |  |  |  |  |  |  |
| **5.** | **I can modify how I work with [the intervention]** |  |  |  |  |  |  |  |  |  |

**SURVEY CONCLUSION**

**Thank you for completing our survey. [ADAPT AS NEEDED]**
